# A Guide to Structured Illumination TIRF Microscopy at High Speed with Multiple Colors

**DOI:** 10.3791/53988

**Published:** 2016-05-30

**Authors:** Laurence J. Young, Florian Ströhl, Clemens F. Kaminski

**Affiliations:** ^1^Department of Chemical Engineering and Biotechnology, University of Cambridge

**Keywords:** Bioengineering, Issue 111, Optical super-resolution, structured illumination microscopy, fluorescence, high speed imaging, TIRF, bioimaging

## Abstract

Optical super-resolution imaging with structured illumination microscopy (SIM) is a key technology for the visualization of processes at the molecular level in the chemical and biomedical sciences. Although commercial SIM systems are available, systems that are custom designed in the laboratory can outperform commercial systems, the latter typically designed for ease of use and general purpose applications, both in terms of imaging fidelity and speed. This article presents an in-depth guide to building a SIM system that uses total internal reflection (TIR) illumination and is capable of imaging at up to 10 Hz in three colors at a resolution reaching 100 nm. Due to the combination of SIM and TIRF, the system provides better image contrast than rival technologies. To achieve these specifications, several optical elements are used to enable automated control over the polarization state and spatial structure of the illumination light for all available excitation wavelengths. Full details on hardware implementation and control are given to achieve synchronization between excitation light pattern generation, wavelength, polarization state, and camera control with an emphasis on achieving maximum acquisition frame rate. A step-by-step protocol for system alignment and calibration is presented and the achievable resolution improvement is validated on ideal test samples. The capability for video-rate super-resolution imaging is demonstrated with living cells.

**Figure:**
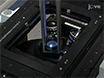


## Introduction

Over the last half a decade, super-resolution microscopy has matured and moved from specialist optics labs into the hands of the biologist. Commercial microscope solutions exist for the three main variants for achieving optical super-resolution: single molecule localization microscopy (SMLM), stimulated emission depletion microscopy (STED), and structured illumination microscopy (SIM)^1,2^. SMLM such as photoactivated localization microscopy (PALM) and stochastic optical reconstruction microscopy (STORM) have been the most popular techniques, largely due to the simplicity of the optical setup and the promise of high spatial resolution, readily down to 20 nm. However super-resolution microscopy *via *single molecule localization comes with an intrinsic trade-off: the spatial resolution attainable is dependent on accumulating a sufficient number of individual fluorophore localizations, hence limiting the temporal resolution. Imaging dynamic processes in live cells therefore becomes problematic as one must adequately sample the movement of the structure of interest to prevent motion artifacts while also acquiring enough localization events in that time to reconstruct an image. In order to meet these requirements, live cell SMLM demonstrations have obtained the required increase in fluorophore photoswitching rates by greatly increasing the excitation power, and this leads in turn to phototoxicity and oxidative stress, thereby limiting sample survival times and biological relevance^3^.

A clear advantage of STED over both SIM and SMLM is that it can image with super-resolution in thick samples, for example lateral resolution of around 60 nm was achieved in organotypic brain slices at depths up to 120 µm^4^. Imaging at such depths with single objective implementations of SMLM or SIM is unfeasible, but becomes possible with either single-molecule light sheet or lattice light sheet microscopy^5^. Video-rate STED has also been demonstrated and used to map synaptic vesicle mobility, although so far this has been limited to imaging small fields of view^6^.

For applications in cell biology and molecular self-assembly reactions^7^^-^^12^ that require imaging with high temporal resolution over many time points, structured illumination microscopy (SIM) can be well-suited as it is not dependent on the photophysical properties of a particular fluorescent probe. Despite this inherent advantage of SIM, up to now its use has been mainly confined to imaging fixed cells or slow moving processes. This is due to the limitations of commercially available SIM systems: the acquisition frame rate of these instruments was limited by the rotation speed of the gratings used to generate the required sinusoidal illumination patterns as well as the polarization maintaining optics. The newest generation of commercial SIM instruments are capable of fast imaging but they are prohibitively expensive to all but central imaging facilities.

This protocol presents a guide to the construction of a flexible SIM system for imaging fast processes in thin samples and near the basal surface of living cells. It employs total internal reflection fluorescence (TIRF) to generate an illumination pattern which penetrates no deeper than approximately 150 nm into the sample^13^ which vastly reduces the out of focus background signal. The idea of combining SIM with TIRF is almost as old as SIM itself^14^ but was not realized experimentally before 2006^15^. The first *in vivo *images obtained with TIRF-SIM were reported in 2009^16^ achieving frame rates of 11 Hz to visualize tubulin and kinesin dynamics, and two color TIRF-SIM systems have been presented^17,18^. Most recently, a guide for the construction and use of a single color two-beam SIM system was presented featuring frame-rates of up to 18 Hz^19,20^.

The set-up presented here is capable of SIM super-resolution imaging at 20 Hz in three colors, two of which can be operated in TIRF-SIM. The whole system is constructed around an inverted microscope frame and uses a motorized *xy* translation stage with a piezo-actuated *z* stage. To generate the sinusoidal excitation patterns required for TIRF-SIM, the system presented uses a ferroelectric spatial light modulator (SLM). Binary grating patterns are displayed on the SLM and the resulting ±1 diffraction orders are filtered, relayed and focused into the TIR ring of the objective lens. The necessary phase shifts and rotations of the gratings are applied by changing the displayed SLM image. This protocol describes how to build and align such an excitation path, details the alignment of the emission path, and presents test samples for ensuring optimal alignment. It also describes the issues and challenges particular to high speed TIRF-SIM regarding polarization control and synchronization of components.

Design Considerations and Constraints

Before assembling the TIRF-SIM system presented in this protocol, there are several design constraints to consider which determine the choice of optical components. All abbreviations of optical components refer to **Figure 1**.

Spatial Light Modulator (SLM)

A binary ferroelectric SLM is used in this setup as it is capable of sub-millisecond pattern switching. Grayscale nematic SLMs may be used but these offer greatly reduced switching times. Each on or off pixel in a binary phase SLM will impart either a π or 0 phase offset to the incident plane wavefront, therefore if a periodic grating pattern is displayed on the SLM it will operate as a phase diffraction grating.

Total Internal Reflection (TIR)

To achieve TIR and produce an evanescent field, the incident angle of the excitation beams at the glass-sample interface must be greater than the critical angle 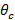. This sets the minimum incident angle required, and hence also the maximum spacing, or period, of the evanescent illumination pattern. The maximum incident angle 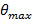 (the acceptance angle) is limited by the numerical aperture (NA) of the objective lens which can be calculated from the definition 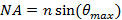. This determines the minimum pattern spacing achievable according to the Abbe formula 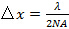 which links NA and wavelength 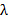 to the minimum pattern spacing 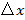. In practice, a 1.49 NA oil immersion TIRF objective yields a maximum angle of incidence of around 79° and a minimum pattern period on the sample of 164 nm using an excitation wavelength of 488 nm. These two angles define a ring in the back aperture of the objective over which the instrument achieves TIR illumination (*i.e*. the TIR ring) and in which the two excitation foci must be accurately positioned and precisely rotated to generate each illumination pattern.

Reconstruction of TIRF-SIM images requires the acquisition of a minimum of three phase shifts per pattern rotation therefore the SLM pattern period must be divisible by 3 (see **Figure 1**). For example, a period of 9 pixels for 488 nm illumination and 12 pixels for 640 nm illumination. For a comprehensive discussion of SLM pattern design, including sub-pixel optimization of pattern spacing using sheared gratings, see the previous work of Kner *et al.*^16^ and Lu-Walther *et al.*^20^ The position of the two excitation foci must be inside the TIR ring for all wavelengths, however the diffraction angle of the ±1 orders from the SLM is wavelength dependent. For standard SIM, multicolor imaging can be achieved by optimizing the grating period for the longest wavelength, and tolerating a loss in performance for the shorter channels. For TIRF-SIM however, optimizing for one wavelength means that the other wavelength foci are no longer within the TIR ring. For example, using a grating period of 9 pixels is sufficient to provide TIRF for 488 nm, as the foci are at 95% of the diameter of the back aperture and within the TIR ring, but for 640 nm this period would position the foci outside the aperture. For this reason different pixel pattern spacings must be used for each excitation wavelength.

The alignment of the TIRF-SIM excitation path is extremely sensitive to small changes in the position of the dichroic mirror (DM4 in **Figure 1**) in the microscope body, much more so than in conventional SIM. Use of a rotating filter cube turret is not recommended, instead use a single, multi-band dichroic mirror, which is kept in a fixed position and designed specifically for the excitation wavelengths used. It is essential that only the highest quality dichroic mirrors are used. These require thick substrates of at least 3 mm, and are often designated as "imaging flat" by manufacturers. All other substrates lead to intolerable aberration and image degradation in TIRF-SIM.

Polarization Control

To achieve TIRF-SIM it is essential to rotate the polarization state of the excitation light in synchronicity with the illumination pattern such that it remains azimuthally polarized in the objective pupil plane with respect to the optical axis (*i.e*. s-polarized). Alignment of the polarization control optics will depend on the specific optical element employed, for example a Pockels cell^21^, or a half wave plate in a motorized rotation stage^22^. In this protocol a custom liquid crystal variable retarder (LCVR) is used, designed to provide full-wave (2π) retardance over the wavelength range 488 to 640 nm as it allows fast (~msec) switching. If using a liquid crystal retarder it is essential to use a high quality component: standard components are typically not stable enough to give a constant retardance over the length of the camera exposure time which leads to a blurring out of the illumination pattern and low modulation contrast. Liquid crystal retarders are also strongly temperature dependent and require built in temperature control.

Synchronization

The lasers must be synchronized with the SLM. Binary ferroelectric SLMs are internally balanced by switching between an on state and off state. The pixels only act as half wave plates in either their on or off state, but not during the interframe switching time. Therefore the lasers should only be switched on during on/off states via the LED Enable signal from the SLM to prevent a reduction in pattern contrast due to the intermediate state of the pixels. An acousto-optic modulator (AOM) could alternatively be used as a fast shutter if the lasers cannot be digitally modulated.

Choice of Lenses

Based on these constraints, the required demagnification of the SLM plane onto the sample plane to produce the desired illumination patterns can be determined. This allows calculation of the focal lengths of the two lenses L3 and L4 in the image relay telescope and the excitation condenser lens L5. In this system a 100X/1.49NA oil immersion objective lens is used with 488 nm and 640 nm excitation, hence uses focal lengths of 300 and 140 mm for L4 and L3, and 300 mm for L5, giving a total demagnification of 357X, equivalent to an SLM pixel size of 38 nm at the sample plane. Using this combination of lenses, SLM grating periods of 9 for 488 nm illumination and 12 pixels for 640 nm give pattern spacings of 172 and 229 nm at the sample, corresponding to angles of incidence of 70° and 67° respectively. For a glass-water interface, the critical angle is 61°, and is independent of wavelength, therefore these two pattern spacings allow TIRF excitation for both wavelengths. An objective lens equipped with a correction collar is useful for correction of spherical aberrations introduced by variations in coverslip thickness, or if operating at 37 °C.

Image Reconstruction

Once raw SIM data has been acquired it is a matter of computational effort to generate super-resolved images in a two-step process. Firstly, the illumination pattern has to be determined for every image and secondly, the components of the SIM spectrum must be separated and recombined appropriately as to double the effective OTF support (see **Figure 6**, insets).

Precise knowledge of the projected illumination patterns is paramount, as the super-resolved frequency components have to be unmixed as accurately as possible to prevent artifacts caused by the residual parts of overlapping components. We determine the illumination pattern parameters *a posteriori* from the raw image data following the procedure introduced by Gustafsson *et al*.^23^ In short, a set of illumination parameters that describes a normalized two-dimensional sinusoid has to be found for each of the 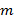 excitation patterns 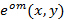:


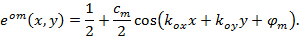


Hereby 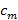 and 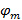 describe the fringe contrast and the pattern starting phase of each individual image m respectively. The components of the wave vector, 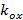 and 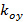 , only change with different orientations 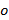 of the pattern and can assumed to be otherwise constant. To coarsely determine the components of the wave vector a cross correlation of raw image spectra is performed, which is refined by applying subpixel shifts to one of the cross-correlated images as to optimize the overlap. This is done via multiplication of real-space phase gradients 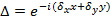 that induce a subpixel shift in frequency-space. Note that it is useful to have a good estimate of the wave-vectors prior to the actual pattern estimation and this can be found by imaging a fluorescent bead layer.

As the phase step between shifted patterns is 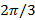, *i.e*. 
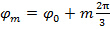
, the separation of frequency components can be performed by a Fourier transform along the "phase axis". The global phase 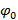 and the fringe contrast 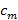 can then be determined using complex linear regression of different components. The individual separated components are then combined using a generalised Wiener filter. For a detailed description of both parameter extraction and implementation of the generalized Wiener filter we refer the reader to Gustafsson *et al.*^23^ where the same algorithm is used.

## Protocol

### 1. Arranging and Aligning the Excitation Path

1. Mark the positions of the components on the optical table (see **Figure 1** for an overview of the optical setup). Separate the objective, lenses L3, L4, L5, and the SLM each by the sum of their respective focal lengths such that the SLM surface will be relayed onto the focal plane of the objective.
2. Insert multi-edge dichroic mirror DM4 into the filter cube turret of the microscope frame.
3. Insert the second dichroic mirror DM3 into a 1" square kinetic mirror mount, and position it one focal length away from the condenser lens L5. Note: This excitation path design incorporates two identical dichroic mirrors DM3 and DM4 which are taken from the same production batch to ensure identical optical properties. The dichroic mirror (DM4) is positioned such that the s- and p- axes are switched compared to the dichroic located in the microscope (DM3) thereby cancelling any polarization ellipticity introduced by its birefringence (**Figure 1**). This compensation works equally well for each illumination wavelength. This step is essential for maintaining high modulation contrast.
4. Before inserting any lenses into the excitation path, accurately define the optical axis for the system.
  1. Remove the objective lens (OB) from the turret and instead screw in an alignment tool. This consists of a 500 mm long optical cage system with two alignment disks at both ends.
  2. Use the dichroic mirror DM3 and a temporary alignment mirror positioned at the approximate later location of the SLM to steer a collimated reference beam from Laser 1 through the center of the holes in the two alignment disks. Direct the beam from Laser 1 to the temporary mirror as depicted in **Figure 1** using three mirrors and dichroic mirror DM2. The temporary mirror at the SLM position must be close to perpendicular to the optical axis. Note: Use Laser 1 as the reference beam, as the other lasers can be subsequently aligned once the excitation path is in place.
  3. Remove the alignment tool once the coarse optical axis has been determined.Insert an iris into the beam path before it enters the microscopy body and center it on the beam. Attach a piece of white card with a small hole centered on the iris.Reinsert the objective lens (OB). Note: The beam leaving the objective will now be highly divergent, but there will be a very weak reflection from the back surface of the lens that will be visible on the white card. All lenses, even if they are anti-reflection coated, will have weak back reflections that can be used to ensure coaxial alignment. If the beam is exactly perpendicular to the lens then the back reflection will go back through the center of the iris
  4. Make iterative angular adjustments to the two mirrors (DM3 and alignment mirror at the SLM position) to center the back reflection on the card with the incoming beam. Temporarily remove the objective lens (OB) and mark the laser spot on the ceiling to create a reference position.
  5. Insert a pair of irises at the height of the reference beam along the threaded holes of the table. The beam should be parallel to the surface of the optical table. The optical axis is now defined.
5. Insert the condenser lens (L5) roughly one focal length away from the objective. Mount this lens on a linear translation stage set to translate along the direction of the reference beam.
6. Adjust the condenser lens position and angle such that the beam leaving the objective is collimated and hits the reference spot on the ceiling. Check that the lens is perpendicular to the beam by again checking the back reflection with the iris and white card. Remove the objective lens (OB) and insert the second lens of the image relay telescope (L4). Note: Ensuring proper collimation and non-deflection of the beam is made easier when there is an even number of lenses in the beam path.
7. Adjust the position and angle of this lens using a linear translation stage to maintain collimation and to ensure the reference beam still hits the marked spot on the ceiling.
8. Replace the objective lens (OB) and insert the first lens of the telescope (L3). Adjust the position and angle of this lens to ensure collimation and non-deflection, as described in previous steps.
9. Mount the SLM chip on a gimbal mount which provides rotation without translation about the center of the chip surface. Note: The specific mounting design depends on the SLM used. If the SLM is supplied without a mount, it should be fixed to a custom machined aluminum plate which is then attached to a lens gimbal mount.
10. With the lenses aligned, insert the SLM in place of the mirror. Adjust the position of the SLM such that the reference beam is located at the center of the SLM chip, and adjust the angle such that the beam passes through the two relay lenses (L3 and L4). Check that the reference beam is still centered on the marked spot.
11. Expand and collimate the reference beam using a Keplerian beam expander.
  1. Mount the two lenses (L1 and L2) in a cage system for ease of adjustment.
  2. Centre the cage system on the reference beam by removing the lenses and replacing them with irises.
  3. Insert the two lenses and adjust the axial position of L2 to collimate the expanded beam using a shearing interferometer. L2 should be one focal length away from the surface of the SLM.
  4. Check that the expanded beam is still collimated after the two relay lenses L3 and L4. Use the shearing interferometer just after DM3 to check for collimation.
12. Once the excitation path has been aligned for a single wavelength, couple the other two lasers into the beam path. Steer each beam through two irises centered on the excitation path using the beam combining dichroic mirrors (DM1 and DM2).

### 2. Alignment of Polarization Rotator

1. Mount the LCVR with its fast axis at 45° to the incident polarization.
2. Fine tune polarization angle of the beam incident to the LCVR using an achromatic half wave plate (HWP) by inserting the HWP and the LCVR between crossed polarizers. Rotate the HWP to minimize the transmitted power. Note: In order to act as a variable polarization rotator, the fast axis of the liquid crystal retarder (LCVR) must be precisely aligned at 45° to the incident vertical beam polarization. The LCVR is physically mounted at 45° but this is only a coarse alignment. The HWP is used to ensure perfect 45° alignment of the incident polarization with respect to the LCVR fast axis. The quarter wave plate (QWP) converts the tilted elliptical polarization induced by the LCVR back to linear polarization at an angle controlled by the applied voltage^24^.
3. Insert the QWP after the LCVR and rotate it to align its slow axis to the incoming polarization by minimizing the transmitted power between crossed polarizers.

### 3. Alignment of the Emission Path

1. Coarsely position the camera using a stage micrometer slide and transmitted light.
  1. Focus on the reticle using the microscope oculars and fix the objective lens at this position.
  2. Roughly center the camera and move the camera position to bring the image of the reticle into focus by observing the image on screen. Note: If an external filter wheel is used then the filter cube will not contain an emission filter, therefore the oculars must not be used when lasers are switched on.
2. Finely adjust the camera position using a fluorescent bead sample.
  1. Prepare a monolayer of fluorescent beads by spreading a drop of 100 nm multicolor beads on a #1.5 coverglass. Leave to dry to adsorb the beads to the coverglass and then re-immerse in water.
  2. Place the bead sample onto the objective with immersion oil. Finely adjust the position of the camera such that the fluorescent bead layer is in focus. Do not adjust the objective lens position once the focus has been found. Note: As the SLM must be in a plane conjugate to the sample plane, the position of the SLM, relay lenses, and objective must be fixed. To adjust the focus, move the sample axially instead of the objective using a piezo z-stage.
3. Generate the appropriate SIM binary grating patterns as bitmap files.
  1. For 2D/TIRF-SIM, generate a series of 9 binary grating images: 3 pattern orientations each with 3 equally spaced phase shifts. Generate these numerically (using MATLAB for example) from a rotated 2D sinusoid with a phase offset applied, then thresholding to produce a binary image. See Supplemental Code Files for example code.
  2. For alignment purposes, also generate grating patterns that have been windowed by a small circular aperture for each of the 3 orientations, as shown in **Figure 2**. The windowed alignment gratings do not need to be externally triggered but can be manually switched by the user via the SLM's software. Note: See references for a discussion of the optimal rotation angles and an example of grating pattern generation code ^16,20^.
4. Upload the bitmap images to the SLM using the manufacturer's software (for example MetroCon).
  1. Load the SLM control software and click "Connect".
  2. In the "Repertoire" tab, click "Load" to open the repertoire file and check the number of Running Orders contained in the file. In the example repertoire file given there are five Running Orders.
  3. Click "Send to Board" to upload the repertoire file to the SLM.
  4. Wait for the bitmap images to upload and for the device to automatically reboot. Note: An example repertoire file, which contains grating bitmap images and a file defining the order, is included as a Supplemental Code File. The ".repz" file may be opened using ZIP file archiver software.
5. Display a windowed alignment grating on the SLM for the first orientation (for example 0°).
  1. In the SLM control software, select the "Status" tab, enter the number of the Running Order (in the case of the example file, this is Running Order "1").
  2. Click "Select" to change the Running Order to the alignment grating. Note: This will illuminate a small circular region in the sample plane. If the SLM surface is correctly conjugated to the sample plane then the edges of this region will be sharply in focus. The grating pattern will produce multiple diffraction orders at the focus of L3: the zero order reflection from the reflective backplane of the SLM, the -1 and +1 orders corresponding to the grating, and also weaker higher orders that arise from diffraction of internal elements specific to the SLM device (*e.g*. reflections of the internal wirings of the SLM pixels and irregularities at the pixel edges). All but the -1 and +1 orders must be filtered out.
6. Insert a spatial mask (SM) mounted in an x,y stage into the beam path at the focal position of L3, and translate its position with respect to the optical axis such that only the desired first orders are passed. Directly after the spatial filter, only two circular beams will be visible. Note: The spatial mask is fabricated by punching 6 holes into aluminum foil using a needle. The holes should be large enough to pass the first order beams for all laser wavelengths. A detailed analysis of the spatial mask is given in reference^20^.
7. Display the next orientation of the alignment grating (60°, running order 2) and again ensure that only the first orders are let through the spatial mask, adjusting its position if required.
8. Repeat for the final orientation (120°, running order 3).
9. Check the image of the fluorescent bead layer on the camera. If the two circular beams are not overlapping as depicted in **Figure 2** then reposition the sample plane by iteratively adjusting the objective lens and camera position.
10. Adjust the objective position to overlap the two beams which will bring the image out of focus. Reposition the camera to bring the image back into focus and fine tune the objective in case two circles are still visible. Repeat this process until the two beams overlap and a single circular region is in focus.
11. Once the position of the sample plane has been set, keep the objective position fixed.
12. To confirm TIRF illumination, image a solution of fluorescent dye, for example for a 488 nm excitation wavelength, use a solution of 10 µM rhodamine 6G.
  1. Bring the dye sample into focus. If the two beams are incident at the correct TIRF angle then single molecules will be visible without high background, and the edges of the circular aperture will be in focus. See **Figure 2B-D** for examples of aligned and misaligned TIRF beams.
  2. Display each orientation of the windowed gratings in turn and ensure that all three orientations provide TIRF illumination and that the two beams overlap at the sample plane. Fine adjustments to the position of the beams can be made by adjusting dichroic mirror DM3. Note: Although different wavelengths are focused at slightly different positions due to axial chromatic aberration, this is not critical and may be corrected by applying a constant z-offset to the sample position prior to excitation with the second wavelength.

### 4. System Synchronization and Calibration

1. Place the bead monolayer sample on the objective and bring into focus.
2. Program the SLM using its control software to display each of the 3 phase shift images in turn, for the first pattern orientation (0°).
  1. Using the SLM control software, switch to Running Order 4 of the example repertoire.
  2. Configure the camera using its acquisition software (for example HCImage) to output two signals: one positive and one negative TTL trigger signal during the global exposure period. In the camera software, under "Advanced Camera Properties", set Output Trigger Kind 1 and 2 to "Exposure", and Output Trigger Polarity 1 and 2 to "Positive" and "Negative" respectively.
  3. Connect Output 1 and 2 of the camera to the "Trigger" and "Finish" inputs of the SLM respectively, using coaxial cables. The SLM is now synchronized to the camera.
3. Acquire a series of 3 images.
  1. In the "Sequence" pane, select "Hard Disk Record" as the scan type, and set the frame count to 3.
  2. Click "Start" to acquire 3 frames. The SLM pattern will change upon each exposure. The fluorescent beads in the image will appear to blink on and off between each of the 3 images. The amount of blinking is a read out of the modulation contrast of the sinusoidal illumination pattern.
4. Rotate the polarization of the excitation laser with the LCVR using custom software in order to achieve azimuthal polarization and therefore the highest modulation contrast for the given pattern orientation.
  1. Load the LCVR calibration software.
  2. Enter 0 and 8 for the Minimum and Maximum Voltage respectively.
  3. Click "Sweep LCVR Voltage" to rotate the polarization. Note: The LCVR retardance is a function of temperature and can drift day-to-day even with temperature control. In this step, optimal azimuthal polarization is found empirically by sweeping the applied voltage between its minimum and maximum voltage which has the effect of rotating the polarization incident at the sample. The modulation contrast is calculated for each voltage^25^ and the voltage that achieves peak contrast is used in the following steps.
  4. Wait for the calibration process to complete, and note down the measured voltage.
5. Repeat this calibration process for the remaining two pattern orientations (60° and 120°) and each of the excitation wavelengths.
6. Synchronize the camera exposure with the LCVR, lasers, emission filter wheel and piezo z-stage^26^. To accomplish this, use a high speed data acquisition (DAQ) board as the master clock source for the system, and use the SLM's LED Enable output signal to modulate the lasers (see **Figure 3B**). Note: The specific implementation is dependent on the components used but the use of a high speed DAQ board for digital trigger synchronization and control of the LCVR using an analog voltage, controlled via software, is recommended. The control software used in this protocol is available upon request.
7. Due to axial chromatic aberration, for each wavelength, also apply a z-offset to the sample stage.
  1. Determine the offset experimentally by focusing on a multicolor bead monolayer sample at the first wavelength (*e.g*. 488 nm) then switching to the second (*e.g*. 640 nm). The beads will now be out of focus.
  2. Refocus the beads and measure the change in z position that was needed. This offset can then be applied to the piezo z-stage every time the excitation wavelength is changed.
8. Using the SLM control software, switch the SLM Running Order to the full series of 9 binary grating images required for TIRF-SIM. This is Running Order 0 in the example repertoire.
9. Using the camera control software, acquire 9 images of the bead sample.
  1. In the "Sequence" pane of the camera software, select "Hard Disk Record" as the scan type, and change the frame count to 9.
  2. Click "Start" to acquire images.
  3. Save the acquired images as TIFF files by selecting "TIFF" as the image type in the "Save Buffered Images" window, and clicking OK.
10. Reconstruct a super-resolution image from the raw TIFF images using commercial or custom software to validate the improvement in resolution over standard TIRF. Note: For our microscope we use custom reconstruction code developed both in-house and by Dr. Lin Shao^27^.

## Representative Results

Multicolor 100nm diameter fluorescent beads were imaged to compare standard TIRF to TIRF-SIM and quantify the attainable improvement in lateral resolution (**Figure 4A**-**B**). Reconstruction of raw frames into super-resolution images was performed using standard algorithms as outlined in the literature^27,28^. It can be seen that TIRF-SIM clearly has significantly higher lateral resolution compared to TIRF. The point spread function (PSF) of a microscope is well approximated by the image of a single sub-diffraction sized fluorescent bead, therefore the PSF and the resolution can be quantified by fitting 2D Gaussian functions to individual beads for each wavelength. The estimated resolution of the microscope based on the mean value of the full width half maximum (FWHM) is 89 nm and 116 nm for 488 and 640 nm TIRF-SIM respectively (**Figure 4C**). This corresponds to a two-fold improvement in lateral resolution for both wavelengths compared to the theoretical diffraction limited case. Fluorescently labelled amyloid fibrils are also an excellent test sample for demonstrating doubled resolution (**Figure 4D**). Amyloid fibrils were formed *in vitro *by incubating β-amyloid labelled with 10% rhodamine derivative dyes (488 nm excitation) for 1 week and subsequently imaging with TIRF-SIM. See reference^12^ for more information.

Subcellular structures with high contrast such as emGFP labelled microtubules (**Figure 5B, G**) or LifeAct-GFP (**Figure 5D**) are ideal for TIRF-SIM imaging and yield high contrast super-resolution images. TIRF-SIM imaging using the setup detailed in this protocol enables observation of a sub-population of microtubules located in the vicinity of the basal cell cortex, and microtubule polymerization and depolymerization can be seen over time (Animated **Figure 1**). Not all samples are amenable to imaging with TIRF-SIM, in particular, low contrast samples without discrete structures. Cells expressing cytosolic GFP lack high resolution information aside from at the edges of the plasma membrane (**Figure 5F**,** H **and** Animated Figure 2**) and are hence sub-optimal for TIRF-SIM imaging as the resulting reconstructions are essentially TIRF images overlaid with artifacts. In such samples, the increase in contrast can often be attributed to the deconvolution step of the reconstruction algorithm.

High modulation contrast is essential for successful SIM imaging. The Fourier transform of the reconstructed image allows visualization of the SIM optical transfer function (OTF) (**Figure 6A**, inset). Without maximizing the modulation contrast for each orientation by ensuring azimuthal polarization with a polarization rotator, there is very little modulation of the high-resolution information in the sample leading to a low signal-to-noise ratio in the SIM passbands. Reconstruction algorithms which use the standard Wiener filter approach will simply amplify the noise in the SIM passbands and yield an image which is essentially a standard TIRF image overlaid with hexagonal (or "honeycomb") ringing artifacts (**Figure 6A**, right panel). A possible enhancement might be the use of iterative^29,30^ or blind reconstruction algorithms^31,32^ to reduce these artifacts depending on the type of sample. We recommend the use of the ImageJ plugin SIMcheck to check the quality of SIM data before and after reconstruction^33^.


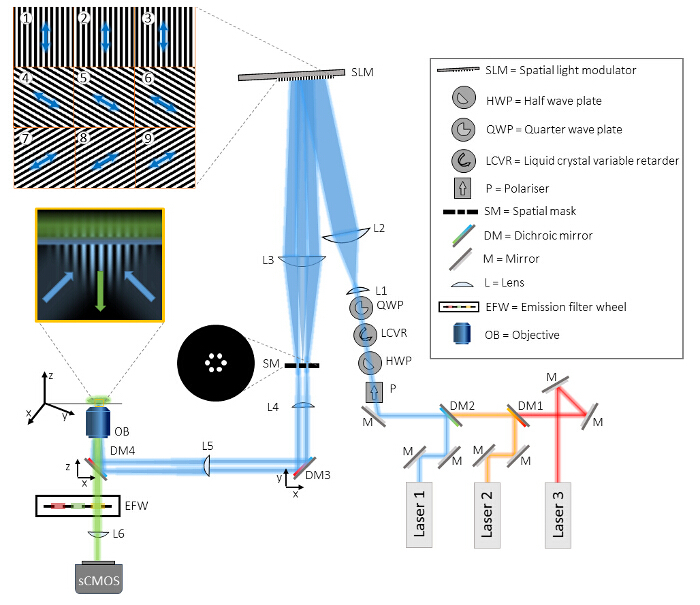
**Figure 1: Layout of the Multicolor TIRF-SIM Setup. **The TIRF-SIM microscope consists of three main parts, the beam generation unit, the pattern projection unit, and the detection unit. In the beam generation unit, three different lasers are aligned onto the same beam path via dichroic mirrors (DM1 and DM2) and directed through four optical elements for polarization control. First, a polarizer (P) ensures the purity of the linear polarization state of each of the laser beams. The following three optical elements are needed to rotate the polarization in a fast, automated manner as described in detail in the text. Afterwards, two lenses (L1 and L2) in a telescope configuration expand the beam to match the active surface of the spatial light modulator (SLM) and are diffracted into three beamlets by the SLM's projected binary grating patterns (examples are shown in tiles 1-9). The polarization state of the illumination light relative to the SLM pattern is shown as an arrow. A second telescope (L3 and L4) de-magnifies the pattern and offers access to the Fourier plane of the SLM pattern. In this plane a spatial mask (SM) is used to filter out the central component and other unwanted diffraction components from the pixelated structure of the SLM and its internal wiring. Before the two remaining beams are focused onto the back focal plane of the objective (OB) via the condenser lens (L5), two dichroic mirrors (DM3 and DM4) are included in the setup. DM4 acts as a conventional dichroic mirror in fluorescence microscopy to separate illumination from emission light. However, this mirror unavoidably induces ellipticity in the polarization state of the illumination light which can be compensated for by DM3, a dichroic mirror from ideally the same batch as DM4. The oil immersion TIRF objective has a large enough NA to directly launch two counter-propagating waves onto the coverslip that are reflected totally and give rise to a structured evanescent field in the coverslip. The sample is mounted on an x-y-z translation stage. Detection is performed through the same objective and DM4 in transmission, plus an additional filtering by bandpass emission filters, mounted in a computer controlled filter wheel (EFW). Finally, the image is projected onto a sCMOS camera by the internal microscope tube lens (L6). Please click here to view a larger version of this figure.


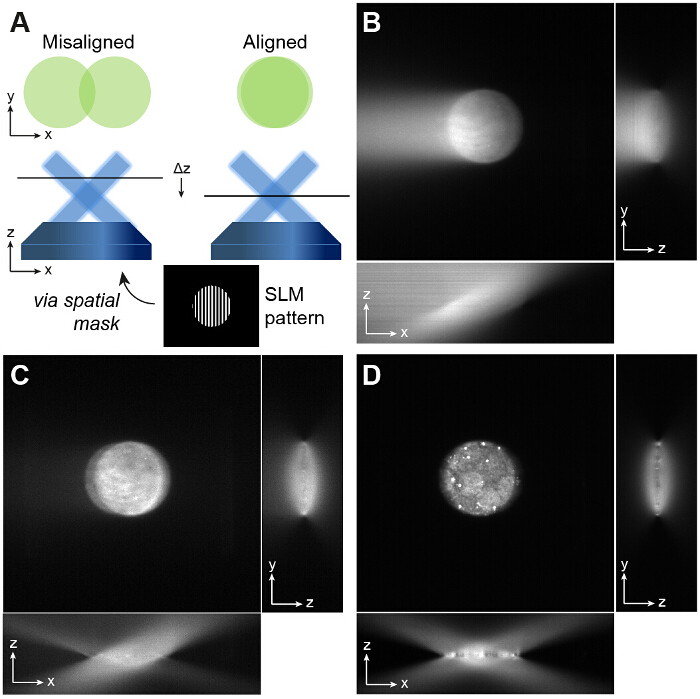
**Figure 2: Alignment of Overlapping Beams.** (**A**) An SLM grating pattern windowed with a circular aperture is useful for alignment. If two non-overlapping beams are visible on the camera (left), then the position of the sample plane must be repositioned by iteratively adjusting the axial positions of the objective lens and the camera to give a single circular illumination spot (right). The beams must overlap in order to produce the sinusoidal excitation pattern required for TIRF-SIM. If the beams do not fully overlap this reduces the field of view over which the interference pattern is formed. (**B** and **C**) The precise angle of incidence of the beams is important for TIRF-SIM. If the angle is incorrect, one of the beams will not be at the required angle for TIRF and this is easily visible when imaging a fluorescent dye solution. One beam has an angle of incidence greater than the critical angle which yields the circular spot, and the other does not, which leads to the bright streak on the left of the image in (**B**). (**D**) Adjusting the angle of mirror DM3 ensures both beams are incident at the same angle, and this can be validated by defocusing the objective: if correctly aligned, the *xz* projection of a *z* stack of a fluorescent dye sample should show two symmetrically intersecting beams with negligible background at the focus. Please click here to view a larger version of this figure.


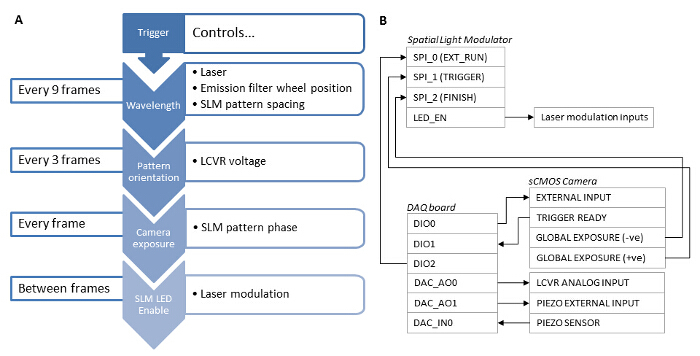
**Figure 3: Synchronization Dependencies of the Different System Components.** (**A**) For fast SIM acquisition, synchronization of the system components using a hardware based solution is essential. (**B**) A data acquisition board (DAQ) should be used as a master trigger. A TTL signal from the DAQ board is sent to the sCMOS External Input and used to trigger the camera exposure. The camera Global Exposure output then triggers the SLM to display a grating pattern, and the SLM LED Enable output is used to digitally modulate the laser excitation such that the laser is only emitting when the SLM pixels are in the "on" state. After the exposure is complete, the camera Global Exposure output is used to advance the SLM pattern on to the next grating phase or angle. The DAQ board also outputs an analog voltage to the LCVR controller to control the linear polarization state of the illumination beam. This voltage is switched after acquisition of the 3 phase images for each pattern angle. After acquisition of 9 images for a single wavelength, the DAQ board outputs a signal to the emission filter wheel controller, and switches to the next wavelength. The DAQ board also applies a z-offset to the sample by outputting an analog voltage to the z-stage piezo controller. Please click here to view a larger version of this figure.


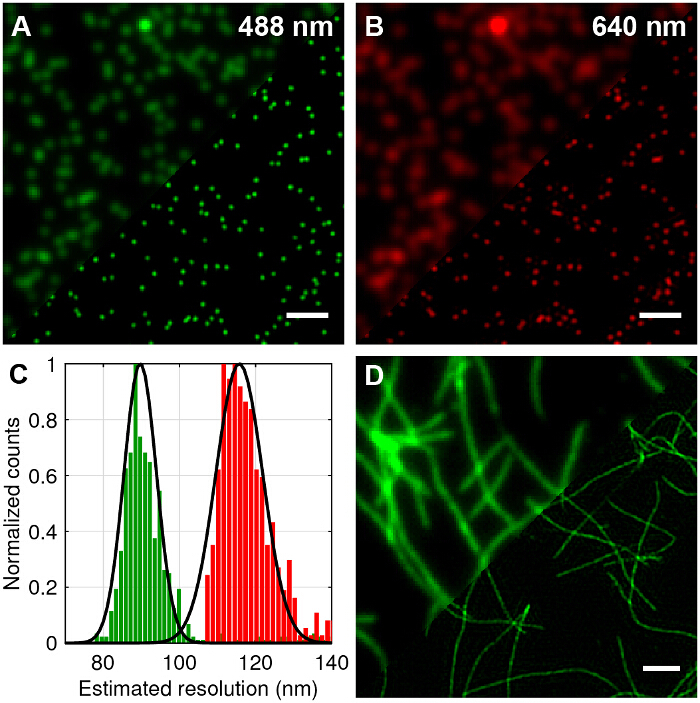
**Figure 4: TIRF-SIM Imaging of Test Samples of 100 nm Multicolor Beads and Fluorescently Labelled Amyloid Fibrils. **(**A** and **B**) Comparison of standard TIRF compared to TIRF-SIM reconstructions for 488 nm and 640 nm excitation. (**C**) Histogram of full-width half-maximum (FWHM) of Gaussian fits to the TIRF-SIM beads showing the expected resolution improvement. (**D**) TIRF versus TIRF-SIM of β-amyloid fibrils labelled with 10% rhodamine derivative dye (488 nm excitation). Scale bars = 1 µm. Please click here to view a larger version of this figure.


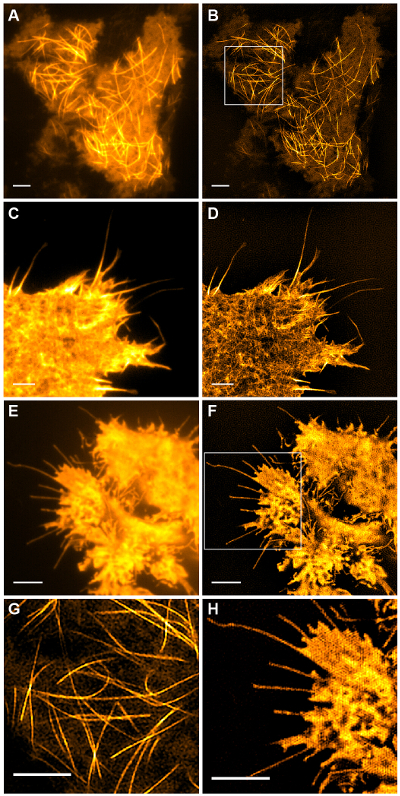
**Figure 5: Live Cell TIRF-SIM Imaging. **Comparison of conventional TIRF and TIRF-SIM images of (**A**, **B**) microtubules (emGFP-tubulin) in a HEK293 cell, (**C**, **D**) filamentous actin (LifeAct-GFP) in a COS-7 cell and (**E**, **F**) cytosolic GFP in a HEK293 cell. Images in B and F are single time points from the movies. Boxed areas are shown magnified in (**G**, **H**). Scale bars = 3 µm. Please click here to view a larger version of this figure.


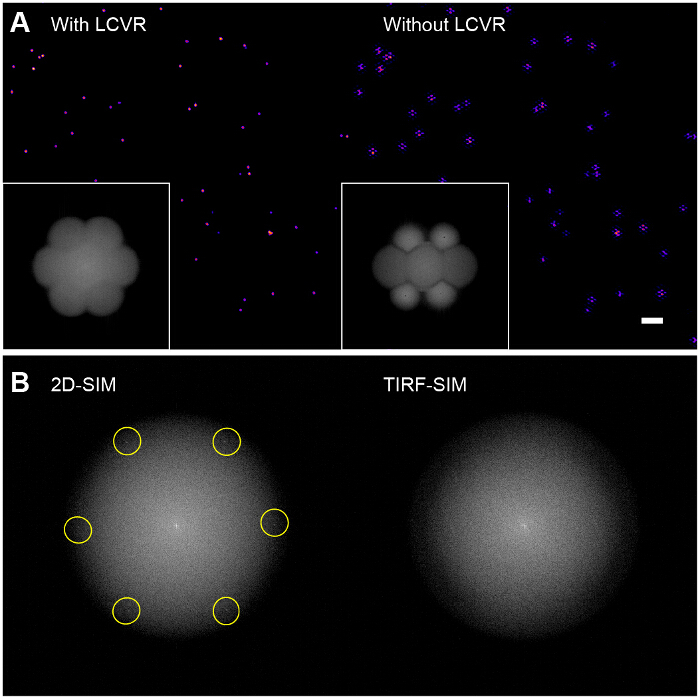
**Figure 6: Influence of Polarization Rotator on Reconstructed Bead Images. **(**A**) Without the use of a polarization rotator such as an LCVR, the signal-to-noise ratio in the SIM passbands is low which results in characteristic hexagonal artifacts in the reconstructed SIM images (right), (**B**) In 2D-SIM, the structured illumination patterns are directly visible in the Fourier transform of the raw images (left, excitation spatial frequency highlighted) as they fall within the radius of the emission OTF support, however in TIRF-SIM, they are outside the OTF support and therefore not visible (right). In this case, the pattern modulation contrast must be assessed using a sparse bead monolayer, as outlined in the protocol. Please click here to view a larger version of this figure.


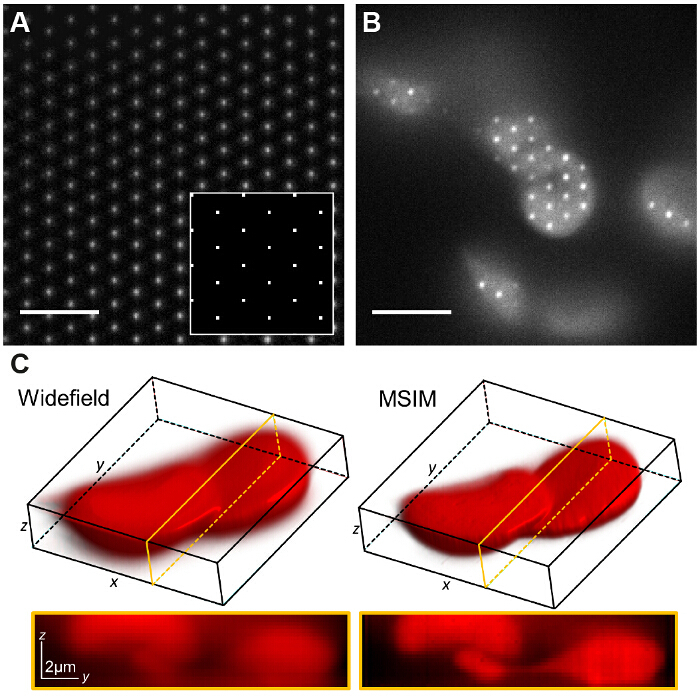
**Figure 7: Spatial Light Modulator Based Pattern Generation Allows Implementation of Other Imaging Modalities Such As Multifocal SIM. **(**A**) In MSIM, a lattice of square pointsdisplayed on the SLM (inset) yields a lattice of diffraction limited foci at the image plane. A thin layer of low concentration rhodamine 6G is imaged to visualize the foci. The pattern is translated across the sample (**B**) and the acquired raw image z-stack is reconstructed to generate an image with reduced out-of-focus light (**C**). Scale bars = 5 µm. Please click here to view a larger version of this figure.


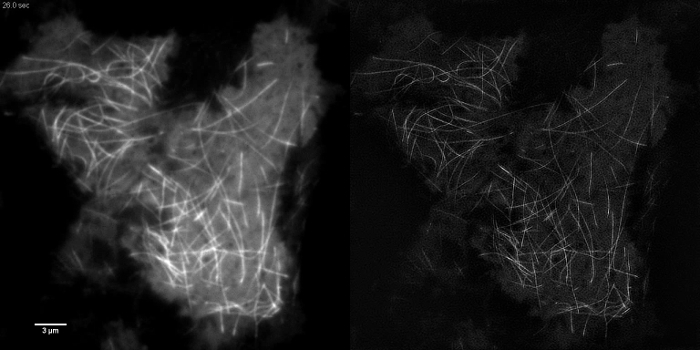
**Animated Figure 1: Time Series Movie of EmGFP-tubulin in a HEK293 Cell. **Rapid polymerization and depolymerization of emGFP labelled microtubules can be observed using TIRF-SIM. Images acquired using 50 msec exposure time per raw frame (450 msec per SIM frame) spaced at intervals of 0.5 sec. Exposure time used was limited by the brightness of the fluorophore, not by the speed of the camera or SLM. Please click here to view this video.


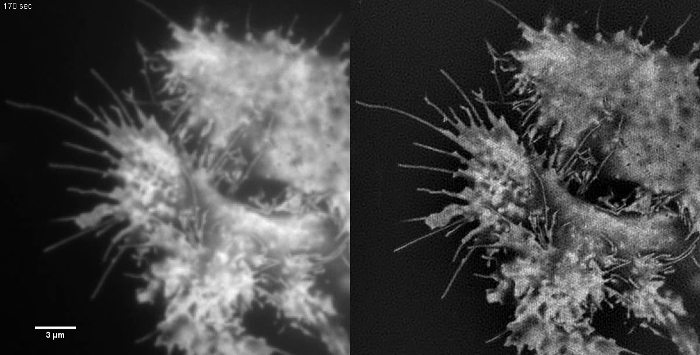
**Animated Figure 2: Time Series Movie of Cytosolic GFP in a HEK293 Cell. **Samples with low contrast such as this are not ideal samples for TIRF-SIM imaging. Retrograde membrane flow can be seen in the TIRF images but TIRF-SIM does not provide any additional information apart from at the cell edges. TIRF-SIM images were acquired using 50 msec exposure time per raw frame (450 msec per SIM frame) spaced at intervals of 5 sec. Please click here to view this video.

**Supplemental Code File:** Example SLM repertoire file (48449_300us_1-bit_Balanced.seq3). Please click here to download this file.

**Supplemental Code File:** Example SLM repertoire file (period9_001.bmp). Please click here to download this file.

**Supplemental Code File:** Example SLM repertoire file (period9_002.bmp). Please click here to download this file.

**Supplemental Code File:** Example SLM repertoire file (period9_003.bmp). Please click here to download this file.

**Supplemental Code File:** Example SLM repertoire file (period9_004.bmp). Please click here to download this file.

**Supplemental Code File:** Example SLM repertoire file (period9_005.bmp). Please click here to download this file.

**Supplemental Code File:** Example SLM repertoire file (period9_006.bmp). Please click here to download this file.

**Supplemental Code File:** Example SLM repertoire file (period9_007.bmp). Please click here to download this file.

**Supplemental Code File:** Example SLM repertoire file (period9_008.bmp). Please click here to download this file.

**Supplemental Code File:** Example SLM repertoire file (period9_009.bmp). Please click here to download this file.

**Supplemental Code File:** Example SLM repertoire file (period9_mask_1.bmp). Please click here to download this file.

**Supplemental Code File:** Example SLM repertoire file (period9_mask_2.bmp). Please click here to download this file.

**Supplemental Code File:** Example SLM repertoire file (period9_mask_3.bmp). Please click here to download this file.

**Supplemental Code File:** Example SLM repertoire file (TIRF-SIM_example.rep). Please click here to download this file.

**Supplemental Code File:** Example grating generation code (1 of 2) (generate_gratings.m). Please click here to download this file.

**Supplemental Code File:** Example grating generation code (2 of 2) (circular_mask.m). Please click here to download this file.

**Supplemental Code File:** Example code to calculate modulation contrast (calculate_contrast.m). Please click here to download this file.

## Discussion

Custom-built TIRF-SIM systems such as the setup detailed in this protocol are capable of multicolor super-resolution imaging at high speed compared to commercially available microscopes. The inherent advantage of SIM as a super-resolution technique is that the temporal resolution is not limited by the photophysics of the fluorophore, compared to other methods such as single molecule localization microscopy (SMLM) or point scanning methods such as stimulated emission depletion microscopy (STED). Unlike these other techniques, SIM does not require photoswitchable or depletable fluorophores so multicolor imaging is straightforward. Non TIRF-SIM systems, such as optical sectioning SIM and multifocal SIM can usually achieve resolution improvements of 1.7 times or less in practice as opposed to the factor of 2 improvement reported here, and commercial systems are also often slower and less flexible than the system presented in this protocol.

The two main difficulties in implementing this technique are firstly the necessity for precise positioning of the six SIM beams within the TIR zone of the objective's back aperture, which requires a laborious and time consuming optical alignment procedure. Secondly, to produce high pattern contrast at the sample, polarization rotation is essential. For low NA 2D-SIM systems, polarization rotation can be avoided by careful choice of the linear polarization orientation, but this becomes impossible for TIRF-SIM^25^. For high-speed multicolor imaging, electro-optical polarization control is necessary and this increases the complexity and expense of the system.

Limitations of the technique

TIRF-SIM, like conventional TIRF, is naturally limited to observation of biological structures and processes located at the basal cell membrane that can be illuminated by the 150-200 nm penetration depth of the evanescent field. While SIM is often quoted as being less photodamaging to cells than either STED or SMLM, lateral resolution doubling does still increase the required number of photons by at least 4-fold^5^ compared to conventional TIRF microscopy. For imaging at high frame rates with short exposures times, this photon increase necessitates use of increased illumination intensities. While any fluorophore may be used for SIM imaging of fixed or slow moving samples, high brightness fluorescent proteins or next generation synthetic dyes with enhanced photostability are recommended for live cell imaging.

Although this implementation is capable of imaging a single color at SIM frame rates in excess of 20 Hz, multicolor imaging in the presented system is limited by the switching time of the motorized emission filter wheel. Due to the large size of the sCMOS camera chip, the use of a multiband emission filter and image splitting optics would be possible and permit simultaneous imaging with multiple wavelengths at no speed penalty. Another possibility would be to alternate the different excitation lasers and use a multiband notch filter to reject the excitation light. The use of a binary ferroelectric SLM in this implementation also is not optimal. The diffraction efficiency of such an SLM is very low, so most of the incident light is in the zero order reflection, which is filtered out by the spatial mask. For applications requiring very high frame rates, the imaging speed is therefore limited by the output power of the laser diodes. The SLM also introduces some ellipticity in the polarization for wavelengths away from the 550 nm design wavelength where the pixels do not operate as ideal half wave plates. Although this could be compensated for by using an additional LCVR, the ideal solution may be the use of a digital micro-mirror device (DMD) as a pattern generator.

Possible modifications

The setup presented here is flexible and more easily modified than commercial instruments so other imaging modalities such as 3D-SIM, fast 2D-SIM, multifocal SIM (MSIM) and non-linear SIM (NL-SIM) can be implemented^21,34,35^.

2D-SIM can be well suited for imaging relatively flat, fast moving structures such as the peripheral endoplasmic reticulum. The peripheral ER lies deeper within the cell than can be illuminated using a TIRF evanescent field but due to its flat structure can be imaged using standard 2D-SIM with negligible out-of-focus background. Additionally, the use of improved optical sectioning reconstruction algorithms to suppress out-of-focus light extend the use of 2D-SIM to optically thick samples, albeit where axial resolution doubling is not required^21^.

In MSIM, the sample is illuminated by a sparse lattice of excitation foci^36^. This modality can be implemented by simply removing the spatial mask (SM) and replacing it by a polarizer. The SLM now operates as an amplitude modulator. The binary SIM gratings displayed on the SLM can be replaced by a 2D lattice of spots, with the size of the spots chosen to be equal to the size of a diffraction limited focus in the image plane. In **Figure 7A**, a lattice of 4 x 4 pixel squares is displayed on the SLM (inset) which when demagnified onto the sample generates diffraction limited foci of 150 x 150 nm, given the physical SLM pixel size of 13.62 µm. The excitation foci can then be translated by shifting the lattice pattern on the SLM and this is repeated multiple times in order to illuminate the entire field of view. Images are acquired for each translated pattern position and the stack is post-processed to yield a reconstructed image with improved resolution of up to a factor of 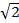 and reduced out-of-focus light compared to the equivalent widefield image^30^. This modality can be useful for imaging thick, dense samples for which standard SIM is unsuited, for example low contrast structures such as stained red blood cells (**Figure 7C**), although the acquisition time is increased due to the large number of raw frames required per field of view (in this case N = 168).

Finally, the setup can be modified to enable either high-NA linear TIRF-SIM or patterned activation non-linear SIM (PA NL-SIM), as presented recently by Li *et al*., by use of an ultrahigh 1.7 NA objective or addition of a 405 nm photoactivation laser and careful optimization of the SLM grating patterns^35^.

Future Applications

SIM is still a rapidly evolving technique and many applications in the life sciences will be enabled in the future. The speed, resolution, and contrast enhancements of the technique and the capability of using standard fluorophores mean that for bioimaging, SIM is set to replace conventional many microscope systems, such as confocal and wide field platforms. Commercial SIM systems are already available today with outstanding technical specifications, however, they are beyond the financial reach of many research laboratories, and, crucially, they are inflexible to be modified and developed to implement the latest research developments in the field. They also lack the essential capability to 'be adapted for the experiment at hand', often a critical bottleneck in cutting edge life science research. The system described here will be particularly well suited to study dynamic processes near the cell surface, for *in vitro *studies of reconstituted bilayer systems, to study surface chemistry in the materials and physical sciences, *e.g*. of 2D materials, and many other applications.

## Disclosures

The authors have nothing to disclose.
